# Nomogram to Predict Poor Outcome after Mechanical Thrombectomy at Older Age and Histological Analysis of Thrombus Composition

**DOI:** 10.1155/2020/8823283

**Published:** 2020-12-17

**Authors:** Longyan Meng, Haichao Wang, Hua Yang, Xiang Zhang, Quanbin Zhang, Qiong Dong, Zhongwen Shu, Liuwei Chen, Li Gong, Yanxin Zhao

**Affiliations:** ^1^Department of Neurology, Shanghai Tenth People's Hospital, Tongji University, Shanghai 200072, China; ^2^Department of Neurosurgery, Binhai People's Hospital, Jiangsu Province, China; ^3^Department of Neurosurgery, Shanghai Tenth People's Hospital, Tongji University, Shanghai 200072, China

## Abstract

An easy scoring system to predict the risk of poor outcome after mechanical thrombectomy among the elderly is currently not available. Therefore, we aimed to develop a nomogram for predicting the probability of negative prognosis in aged patients with acute ischemic stroke undergoing thrombectomy. In addition, we sought to investigate the association between histological thrombus composition and stroke characteristics. To this end, we prospectively studied a developed cohort using data collected from a stroke center from November 2015 to December 2019. The main outcome was functional independence, defined as a modified Rankin Scale score ≤ 2 at 90 days following a mechanical thrombectomy. A nomogram model based on multivariate logistic models was generated. The retrieved thrombi were stained with hematoxylin and eosin and assessed according to histological composition. Our results demonstrated that age ≥ 72 years was independently associated with poor outcome. A total of 304 participants completed the follow-up data to generate the nomogram model. After multivariate logistic regression, five variables remained independent predictors of outcome, including older age, hemorrhagic transformation, thrombolysis in cerebral infarction score, National Institute of Health Stroke score, and neutrophil-to-lymphocyte ratio, and were used to generate the nomogram. The area under the receiver-operating characteristic curve of the model was 0.803. The clots from elderly subjects with large-artery atherosclerosis, anterior circulation, and successful recanalization groups had a higher percentage of fibrin compared to those of younger patients. This is the first nomogram to be developed and validated in a stroke center cohort for individualized prediction of poor outcome in elderly patients after mechanical thrombectomy. Clot composition provides valuable information on the underlying pathogenesis of oxidation in older patients.

## 1. Introduction

Stroke is a leading cause of serious long-term disability and mortality in China, and its incidence increases with age [1]. Advanced age is particularly related to poor poststroke functional outcomes. Mechanical thrombectomy is an effective therapy for patients with acute ischemic stroke (AIS) due to large vessel occlusions [2, 3]. Although the latest guidelines do not recommend an upper age limit for mechanical thrombectomy, there are increasing challenges with endovascular thrombectomy in the elderly, as well as the need to consider comorbidities in clinical decision-making [4, 5]. A high neutrophil-to-lymphocyte ratio (NLR) indicates high neutrophil counts and/or low lymphocyte counts and is considered to accurately reflect the negative effects of neutrophils, as well as the positive roles of lymphocytes in patients with stroke [6–8]. Our previous findings have suggested that histological thrombus composition might provide valuable information on stroke etiology [3], but its relation to outcomes after mechanical thrombectomy in older patients is less well established.

Nomograms are useful tools for clinicians to make a visualized and quick risk assessment and have been widely used for clinical decision-making in patients [9, 10]. However, nomograms have only recently been applied to predict outcomes after mechanical thrombectomy [11], and further validation is necessary among the elderly. The main objective of this study was to develop a nomogram model to predict poor outcomes after mechanical thrombectomy at an older age and to compare the diversity of histological thrombus composition.

## 2. Materials and Methods

### 2.1. Study Design, Participants, and Patient Consent

This was a longitudinal, prediction model development and validation study, which included data from a stroke center from November 2015 to December 2019. Participants were enrolled if they met the following criteria: age ≥ 18 years, with anterior or posterior circulation stroke, irrespective of National Institute of Health Stroke (NIHSS) score at presentation and whether intravenous thrombolysis was performed. Patients were excluded if they were treated with intravenous thrombolysis only, missed key outcome data, or had follow-up loss at 3 months. The study was approved by the Ethics Committee of Shanghai Tenth People's Hospital (Shanghai, China) and was conducted in accordance with the principles of the Declaration of Helsinki. All participants and their caregivers provided written informed consent.

### 2.2. Outcome

The main outcome was functional independence, defined as a modified Rankin Scale (mRS) score ≤ 2 90 days following a mechanical thrombectomy.

### 2.3. Predictors and Clot Histological Analysis

Demographic and clinical data and procedural characteristics were collected at admission, as in our previous study [3]. Stroke subtypes were determined in accordance with the Trial of ORG10172 in Acute Stroke Treatment classification [12], and successful reperfusion was defined as a thrombolysis in cerebral infarction (TICI) score of 2b-3. Symptomatic intracranial hemorrhage (sICH) was defined as any type of ICH with an increase of ≥4 NIHSS score points from baseline within 24 h or leading to death [13].

As described in our previous study, all formalin-fixed, paraffin-embedded thrombi were sectioned and stained with hematoxylin-eosin [3]. The percentages of red blood cells (RBCs), white blood cells (WBCs), and fibrin in retrieved thrombi were analyzed by two experienced pathologists (LJ.F and WZ) who were blinded to the clinical data and imaging findings.

### 2.4. Follow-Up

A total of 322 patients enrolled in the present study were scheduled for an outpatient follow-up at 3 months. Follow-up of the mRS at 3 months was conducted by telephone or at a stroke follow-up clinic. Finally, 302 subjects (93.8%) took part in the follow-up visit. The remaining 20 subjects were lost to follow-up, among whom 12 had travelled out of Shanghai and 8 refused follow-up.

### 2.5. Statistical Analysis

Descriptive statistics and univariate comparisons were used for statistical analysis. Comparisons were performed using Student's *t* test or analysis of variance with Bonferroni's multiple comparisons for continuous measures, the nonparametric *t* test or Mann-Whitney test with Dunn's multiple comparisons for noncontinuous variables, and the *χ*^2^ test with likelihood ratios for categorical measures. All tests were two-sided, and a *P* value < 0.05 was considered significant. Multivariate logistic regression analyses were used to predict good outcome and mortality (two independent models) of patients with a complete dataset. All multivariate models were assessed using the Hosmer-Lemeshow test and *c*-statistics. Variables were included in the models on the basis of statistical significance on univariate analysis and/or clinical relevance. To generate the nomogram, multivariate logistic regression analysis was performed to evaluate the strength of the aforementioned association according to the odds ratio (OR) and corresponding 95% confidence interval (CI) using the forward Wald method; the *F* probability of entry was set at 0.05 and that of removal was set at 0.10. Variables with *P* values < 0.05 were incorporated into the R language to establish the nomogram of the prediction model. The area under the receiver-operating characteristic curve (AUC-ROC) was used to calculate the predictive accuracy of the nomogram model. Then, the nomogram was subjected to bootstrapping validation (1,000 bootstrap resamples) to calculate a relatively corrected *C*-index, which is equivalent to the AUC-ROC. Finally, calibration curves were plotted to assess the calibration of the nomogram by comparing the observed probability of negative outcome after endovascular thrombectomy according to the total score of the nomogram against the predicted probability based on the nomogram. A significant test statistic implied that the model was calibrated perfectly. The Hosmer-Lemeshow test was used to evaluate whether the observed event rates matched the expected rates. Statistical analyses were performed using SPSS v26 (IBM Corporation, New York, USA) and GraphPad Prism 7 (GraphPad Software, La Jolla, CA, USA).

## 3. Results

### 3.1. Characteristics of the Development Cohort

A total of 322 patients who met the inclusion criteria completed the follow-up visit at 90 days. The mean age of the patients was 69 ± 11 years, and 43.4% were women. The average symptom onset-to-groin time was nearly 422 min, and the mean NIHSS score at presentation was 14.4 ± 4.6. Posterior circulation stroke was treated in 18.2% of the patients. Successful reperfusion with TICI ≥ 2b was reported in 87.7% of subjects, and the overall rate of sICH was 31%. A good outcome (mRS score, 0–2) was observed in 39.1% of the cases at 90 days, and the overall mortality at 90 days was 24.4%.

The ROC correlating age and probability of poor outcomes showed optimal sensitivity and specificity trade-off at the age of 72 years. Therefore, patients were grouped by age according to the cut-off point of 72 years, and the elderly were defined as those aged ≥ 72 years. The average age in the elderly group was 79 ± 5 years compared with 61 ± 8 years in the younger population. Univariate analysis showed a significantly higher percentage of women in the elderly group (65% vs. 28%, *P* < 0.01). Elderly patients had a significantly higher incidence of atrial fibrillation (60.9% vs. 37.4%, *P* < 0.01). A slightly higher NIHSS score on admission was found in the elderly population compared with younger adults (14 ± 5 vs. 15 ± 4, *P* = 0.01). There was no significant difference in the rates of posterior circulation strokes, postprocedural sICH, or treatment of intravenous thrombolysis between the two groups. For clinical outcomes, the median mRS score at 90 days was 4 in the elderly group and 3 in the younger group, and the rates of good outcomes (mRS score, 0–2) were 28.1% and 47.4%, respectively (*P* = 0.0001) (Table 1).

### 3.2. Multivariate Analysis of the Development Cohort

In multivariate regression analysis, age ≥ 72 correlated with significantly higher odds of poor outcome (OR = 2.25, *P* = 0.012) but was not associated with higher odds of mortality at 90 days. Additional significant predictors of poor outcomes included higher admission NIHSS scores, lower TICI scores, postprocedural sICH, diabetes, and a higher baseline NLR (Table 2).

### 3.3. Subgroup Analysis for Outcome Predictors

To determine if a specific clinical subgroup of subjects was associated with a higher risk of poor outcome, we performed multivariate analysis to predict an mRS score ≥ 2 at 90 days on the basis of stroke location, the extent of recanalization (TICI score), and stroke subtypes. When patients were grouped by location (anterior or posterior circulation stroke), age at presentation of ≥72 was associated with lower odds of good outcomes in the anterior circulation (OR = 2.52, *P* = 0.01), but not in posterior circulation (Table 3). When stratified by stroke subtype (large-artery atherosclerosis or cardioembolism), an age at presentation of ≥72 correlated with higher odds of poor outcomes in patients with cardioembolic stroke but not in those with large-artery atherosclerosis (LAA) (OR = 5.42, *P* = 0.002) (Table 4). Similarly, when patients were grouped according to TICI scores (TICI < 2b or TICI ≥ 2b), an age of ≥72 years at presentation was associated with higher odds of poor outcome in patients with TICI ≥ 2b (OR = 2.02, *P* = 0.032) (Table 5). Additionally, when patients were grouped according to stroke location, subtype, and etiology, a high NLR showed an independent association with a higher likelihood of poor prognosis.

### 3.4. Development of an Individualized Prediction Model

**Figure 1:**
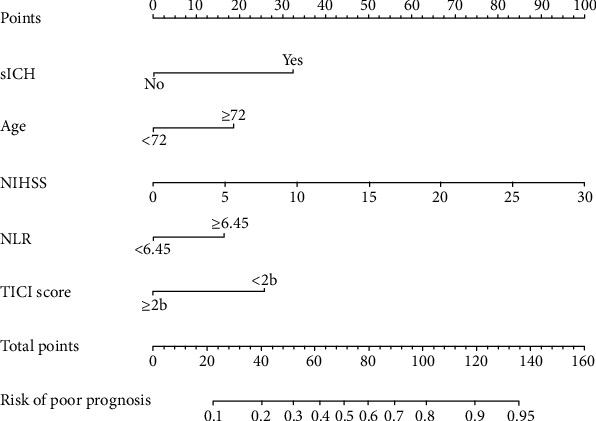
Nomogram for predicting the probability of poor outcome after mechanical thrombectomy. NIHSS: National Institute of Health Stroke; NLR: neutrophil-to-lymphocyte ratio; TICI: thrombolysis in cerebral infarction; ICH: intracerebral hemorrhage.

Hypertension and diabetes did not achieve significance as variables in the binary logistic regression model and were eliminated. Five potential predictors yielded by the binomial logistic regression model (LR method) included age ≥ 72 (OR = 2.25; 95% CI, 1.20–4.25), TICI score (OR = 0.32; 95% CI, 0.11–0.91), NIHSS score (OR = 1.16; 95% CI, 1.08–1.24), sICH (OR = 4.9; 95% CI, 2.54–9.45), and NLR (OR = 2.27; 95% CI, 1.27–4.07). A prediction model was established using the nomogram based on these five factors, and a summary of the point value of each factor used to calculate the total score is presented in Figure 1. The AUC-ROC curve of the prediction model was 0.803, with a sensitivity of 67.9% and specificity of 82.8%.

### 3.5. Performance of the Nomogram to Predict Negative Outcomes after Endovascular Thrombectomy at Older Age

**Figure 2:**
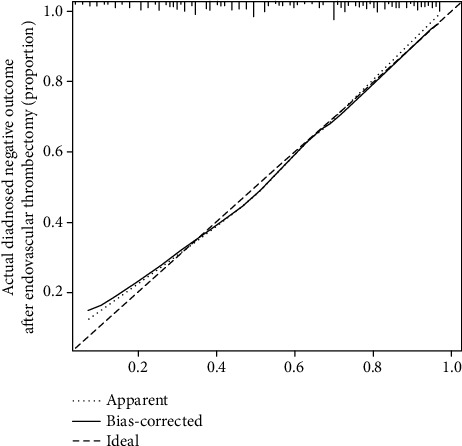
Nomogram-predicted probability of negative outcome after mechanical thrombectomy.

The calibration curve of the nomogram for the predicted probability of negative outcome after endovascular thrombectomy at older age in this cohort is shown in Figure 2. The results of the 1,000 bootstrap samples estimated the AUC to be 0.791, which suggested good discrimination of the model.

### 3.6. Histological Analysis of Clot Composition

**Figure 3:**
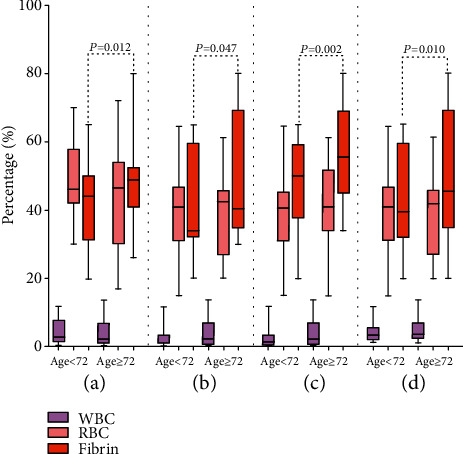
(a) Thrombi composition in patients aged ≥72 and <72 years; (b) thrombi composition in patients aged ≥72 and <72 years with anterior circulation stroke; (c) thrombi composition in patients aged ≥72 and <72 years with large-artery atherosclerosis stroke; and (d) thrombi composition in patients aged ≥72 and <72 years with successful recanalization (TICI ≥ 2b). WBC: white blood cell; RBC: red blood cell.

Of the 147 materials collected from the occlusion artery, clots were classified as cardioembolism and LAA. As shown in Figure 3(a), the fibrin composition differed significantly between the patients aged ≥72 and <72years (48.7% vs. 43.8%, *P* = 0.012). Furthermore, the clots from the elderly subjects in the LAA (42.0% vs. 39.5%, *P* = 0.002), anterior circulation (58.3.0% vs.52.6.5%, *P* = 0.047), and successful recanalization (44.8% vs. 41.0%, *P* = 0.01) groups had a higher percentage of fibrin compared with younger patients (Figures 3(b)–3(d)).

## 4. Discussion

Our findings suggest that an older age of ≥72 years is independently associated with poor outcomes despite comparable clinical characteristics and procedural variables. We present a precise nomogram model based on older age, hemorrhagic transformation, the TICI score, the NIHSS score, and NLR to predict the probability of poor outcomes following mechanical thrombectomy. This model provides clinicians with a useful tool for quick and individualized preoperative risk assessment in elderly patients aged ≥72 years.

The negative prognostic effect of older age persisted despite anterior circulation, cardioembolic etiology, and successful recanalization (TICI ≥ 2b). We demonstrate that an age of ≥72 years is a negative predictor of poor outcome in patients with anterior circulation occlusion but does not independently predict an increased rate of functional dependence in the posterior circulation group. Our findings and those of previous studies have consistently shown that age is an independent predictor of functional independence and mortality after thrombectomy in elderly patients with acute anterior circulation stroke [14, 15]. Although the BASICS study demonstrated that mechanical thrombectomy is safe for acute circulation stroke, its effectiveness has not yet been confirmed [16]. There is new evidence suggesting that mechanical thrombectomy of cardioembolic large-vessel occlusion has a higher rate of successful reperfusion and higher efficacy compared with those of noncardioembolic etiology [17]. Our data suggest an independent association of age ≥72 years with poor outcome despite cardioembolic etiology, which raised concerns about preoperative risk assessment. Consistent with existing findings, older age may predict functional dependence after mechanical thrombectomy in patients with AIS despite successful recanalization [18]. These data seem to suggest that it might be difficult for elderly patients with severe neurological impairment at baseline to undergo a successful procedure. In addition to older age, a higher NLR on admission was a strong modifiable predictor of poor outcome when patients were divided according to stroke location, etiology, or extent of recanalization (TICI scores). The NLR is a potential novel biomarker of oxidative stress, which has recently been reported as an important predictor of clinical outcome after mechanical thrombectomy [19]. Our study further strengthens the importance of the NLR in predicting clinical outcomes after mechanical thrombectomy.

An important result in the present study was the establishment of a nomogram model to predict functional outcome and mortality, as this has not been reported previously among survivors aged ≥ 72 years. This model showed good discrimination and calibration, indicating that the predictive power is as good as representing actual risk. The verification of the internal validation group (training group) by bootstrap analysis further confirmed the reliability of the present nomogram. Although several studies have examined the relationship between risk predictors and stroke outcome after mechanical thrombectomy [14, 15, 20], there is growing evidence that nomograms have a better performance than risk predictors [10, 11]. In contrast to the risk group, a nomogram model provides a visualized and individualized estimate of the prediction probability of a specific outcome for an individual patient, as well as an important tool of medical decision-making based on the individual's disease characteristics. Thus, this model, showing good predicting ability, can be used by clinicians to increase confidence in their preprocedure risk assessment.

Although our primary goal was to develop a nomogram model to predict poor outcome after mechanical thrombectomy at an older age, we also investigated differences in the histological characteristics of thrombi between the two age groups. Our results demonstrate that age at presentation ≥72 years was associated with a higher proportion of fibrin in thrombi than those <72 years in anterior circulation, cardioembolic etiology, and successful recanalization. Increased plasma fibrin levels have been reported to be associated with a significantly higher risk of major stroke events in aged patients [21]. In the present study, we measured plasma fibrin in thrombi and found that fibrin-rich thrombi were more common in older patients. Fibrin-rich thrombi may have deleterious effects on the development of stroke by several mechanisms, including elevated friction between the clot and the vessel wall, lower successful recanalization rates, and increased number of passes at retrieval, and subsequent severe endothelial damage, all of which may lead to catastrophic outcomes and higher risk of mortality [22–24]. Moreover, oxidative stress has been reported to correlate with unfavorable changes in fibrin clot properties, including faster formation of tough thrombus and lower successful recanalization rates [25–27]. Thus, fibrin-rich thrombi may be a potential oxidative marker of older patients with large vessel occlusion undergoing mechanical thrombectomy.

The major strengths of our study include the development of a readily available and rapid tool, which can facilitate individual preprocedure identification of negative outcomes at older age, as well as homogeneous histological analysis in the included patients. However, this study has some limitations. First, this study is a single-center, observational study with a natural bias, and if possible, an external validation in a multicenter cohort with a larger patient number should be considered. Second, the relationship between functional outcome and plasma NLR may be dynamic and may be influenced by multiple variables such as diet or fever, which may have influenced the results. The dynamic NLR data will be considered in future studies because of its more effective predicting ability. Finally, the components of the retrieved thrombi might not completely reflect those of the entire thrombus, and although we attempted to exclude this unavoidable bias, this influence should be considered.

## 5. Conclusion

This study provides an easy-to-use nomogram model to predict poor outcomes after mechanical thrombectomy at an older age of ≥72 years. This nomogram provides clinicians with a new tool that is more useful for the visualization of risk for each individual than traditional risk scores in the preprocedure decision-making process. In addition, we found more fibrin-rich thrombi among elderly survivors undergoing mechanical thrombectomy, suggesting that oxidative stress links older age to neurological dysfunction. Future studies are warranted to investigate this potential target in elderly stroke patients undergoing mechanical thrombectomy.

## Figures and Tables

**Table 1:** Comparison of baseline characteristics and procedural and outcome measures in patients aged <72 years and those aged ≥72 years.

Baseline characteristics	Age (years)	Age (years)	Test summary
	<72 (*n* = 174)	≥72 (*n* = 128)	
Sex (female)	48 (27.6%)	83 (64.8%)	0.0001
Age, years (SD)	61.94 (7.51)	79.50 (4.59)	0.0001
NIHSS on admission, median (range)	13.82 (4.70)	15.28 (4.39)	0.006
*Location*			
Anterior circulation	136 (78.2%)	111 (86.7%)	0.057
Posterior circulation	38 (21.8%)	17 (13.3%)	
IV tPA	74 (42.5%)	41 (32.0%)	0.063
*Vascular risk factors*			
Hypertension	106 (60.9%)	90 (70.3%)	0.091
Diabetes mellitus	42 (24.1%)	29 (22.7%)	0.764
Atrial fibrillation	65 (37.4%)	78 (60.9%)	0.0001
Coronary heart disease	17 (9.8%)	26 (20.3%)	0.010
*Laboratory findings*			
Hemoglobin	129.47 (19.19)	118.30 (20.26)	0.0001
WBC, mean (SD)	9.03 (2.96)	9.41 (4.41)	0.366
Neutrophilic	8.28 (7.92)	8.29 (7.22)	0.365
Lymphocyte	1.51 (3.41)	1.22 (1.60)	0.365
NLR	9.23 (7.23)	9.69 (8.75)	0.614
NLR ≥ 6.45	98 (56.3%)	74 (57.8%)	0.796
C-reactive protein, mean (SD)	20.60 (37.81)	21.21 (33.94)	0.883
Fibrinogen (SD)	2.82 (0.83)	3.11 (0.92)	0.006
D-Dimer, mean (SD)	3.29 (7.45)	3.96 (5.58)	0.397
Median onset-to-groin time, min (SD)	300.85 (544.50)	268.95 (138.11)	0.518
Median time from symptom onset to vascular recanalization, min (SD)	328.11 (172.76)	335.60 (152.71)	0.697
*Outcome*			
Reperfusion after endovascular intervention TICI (2b/3)	159.76 (8%)	109 (85.2%)	0.239
ICH	57 (32.8%)	43 (33.6%)	0.879
Clinical outcome mRS score (0–2)	82 (47.4%)	36 (28.1%)	0.001
Mortality	26 (15.0%)	36 (28.1%)	0.005

NIHSS: National Institute of Health Stroke; IV tPA: intravenous recombinant tissue-type plasminogen activator; NLR: neutrophil-to-lymphocyte ratio; TICI: thrombolysis in cerebral infarction; ICH: intracerebral hemorrhage; WBC: white blood cell.

**Table 2:** Multivariate analysis for predictors of poor outcome and mortality 90 days after endovascular thrombectomy.

Variable	Multivariate model for poor outcome (*n* = 302)			Multivariate model for mortality (*n* = 302)		
	OR	95% CI	*P* value	OR	95% CI	*P* value
Sex (female)	1.54	0.84 to 2.82	0.161	1.33	0.63 to 2.79	0.450
Age ≥ 72 years	2.25	1.20 to 4.25	0.012	1.87	0.89 to 3.95	0.099
Onset-to-groin time	1.00	0.99 to 1.01	0.162	1.00	0.99 to 1.00	0.711
Location	1.54	0.69 to 3.48	0.292	1.32	0.54 to 3.20	0.542
Hypertension	1.75	0.95 to 3.24	0.075	1.61	0.73 to 3.52	0.237
Diabetes mellitus	2.34	1.16 to 4.70	0.017	3.59	1.69 to 7.60	0.001
Atrial fibrillation	0.80	0.44 to1.47	0.469	1.09	0.53 to 2.23	0.823
Coronary heart disease	0.78	0.34 to1.77	0.558	1.20	0.48 to 2.99	0.700
TICI score	0.32	0.11 to 0.91	0.032	0.26	0.11 to 0.63	0.003
NIHSS score	1.16	1.08 to 1.24	0.0001	1.20	1.10 to 1.31	0.0001
IV tPA	0.94	0.51 to 1.73	0.839	1.08	0.52 to 2.24	0.844
ICH	4.90	2.54 to 9.45	0.0001	2.33	1.17 to 4.64	0.016
NLR ≥ 6.45	2.27	1.27 to 4.07	0.006	3.62	1.66 to 7.90	0.001

NIHSS: National Institute of Health Stroke; IV tPA: intravenous recombinant tissue-type plasminogen activator; NLR: neutrophil-to-lymphocyte ratio; TICI: thrombolysis in cerebral infarction; ICH: intracerebral hemorrhage; OR: odds ratio; CI: confidence interval.

**Table 3:** Multivariate analysis for predictors of poor outcome and mortality 90 days after endovascular thrombectomy by location.

	Multivariate anterior circulation (*n* = 247)			Multivariate posterior circulation (*n* = 55)		
Variable	OR	95% CI	*P* value	OR	95% CI	*P* value
Sex (female)	1.39	0.72 to 2.70	0.327	2.81	0.50 to 15.78	0.240
Age ≥ 72 (years)	2.52	1.25 to 5.07	0.010	0.42	0.06 to 3.16	0.398
Onset-to-groin time	1.00	0.99 to 1.00	0.483	1.01	0.99 to 1.02	0.105
Hypertension	1.81	0.91 to 3.60	0.092	7.53	0.72 to 78.43	0.091
Diabetes mellitus	2.16	0.97 to 4.80	0.059	4.53	0.69 to 29.58	0.115
Atrial fibrillation	0.93	0.48 to 1.82	0.834	0.26	0.05 to 1.355	0.110
NIHSS score	1.15	1.06 to 1.25	0.001	1.28	1.05 to 1.57	0.016
IV tPA	0.83	0.43 to 1.60	0.568	2.20	0.29 to 16.68	0.445
ICH	5.40	2.66 to 10.96	0.0001	3.31	0.39 to 27.40	0.268
NLR ≥ 6.45	2.15	1.12 to 4.12	0.021	8.80	1.14to 68.00	0.037

NIHSS: National Institute of Health Stroke; IV tPA: intravenous recombinant tissue-type plasminogen activator; NLR: neutrophil-to-lymphocyte ratio; TICI: thrombolysis in cerebral infarction; ICH: intracerebral hemorrhage; OR: odds ratio; CI: confidence interval.

**Table 4:** Multivariate analysis for predictors of poor outcome and mortality 90 days after endovascular thrombectomy by stroke subtype.

Variable	Multivariate model for LAA (*n* = 159)			Multivariate model for CE (*n* = 143)		
	OR	95% CI	*P* value	OR	95% CI	*P* value
Sex (female)	1.97	0.81 to 4.80	0.136	1.85	0.69 to 4.98	0.224
Age ≥ 72 (years)	1.04	0.42 to 2.57	0.932	5.42	1.84 to15.95	0.002
Onset-to-groin time	1.00	0.99 to 1.00	0.220	1.00	0.99 to 1.01	0.467
Location	2.85	1.03 to 7.87	0.043	0.39	0.08 to 2.01	0.263
Hypertension	2.37	1.00 to 5.59	0.050	1.57	0.55 to 4.49	0.400
Diabetes mellitus	2.11	0.87 to 5.13	0.099	5.12	1.19 to 22.07	0.029
Coronary heart disease	0.82	0.24 to 2.84	0.750	0.78	0.19 to 3.21	0.726
TICI score	0.21	0.05 to 0.91	0.037	0.220	0.03 to 1.47	0.119
NIHSS score	1.12	1.02 to 1.23	0.016	1.28	1.12 to 1.46	0.0001
IV tPA	2.10	0.84 to 5.28	0.114	0.41	0.14 to 1.18	0.097
ICH	2.73	1.08 to 6.92	0.035	11.100	3.58 to 34.38	0.0001
NLR ≥ 6.45	2.34	1.00 to 5.44	0.049	4.53	1.55 to 13.23	0.006

NIHSS: National Institute of Health Stroke; IV tPA: intravenous recombinant tissue-type plasminogenactivator; NLR: neutrophil-to-lymphocyte ratio; TICI: thrombolysis in cerebral infarction; ICH: intracerebral hemorrhage; OR: odds ratio; CI: confidence interval; LAA: large-artery atherosclerosis; CE: cardioembolism.

**Table 5:** Multivariate analysis for predictors of poor outcome and mortality 90 days after endovascular thrombectomy by TICI score.

Variable	Multivariate TICI ≥ 2b (*n* = 265)		
	OR	95% CI	*P* value
Sex (female)	1.38	0.74 to 2.57	0.31
Age ≥72	2.02	1.06 to 3.84	0.032
Onset-to-groin time	1.00	0.99 to1.00	0.363
Location	1.49	0.67 to 3.33	0.333
Hypertension	1.71	0.91 to 3.20	0.095
Diabetes mellitus	2.55	1.25 to 5.22	0.01
Atrial fibrillation	0.86	0.46 to 1.61	0.64
Coronary heart disease	0.86	0.37 to 1.98	0.726
NIHSS score	1.15	1.07 to 1.23	0.0001
IV tPA	1.01	0.54 to 1.90	0.979
ICH	4.44	2.25 to 8.75	0.0001
NLR ≥ 6.45	2.29	1.26 to 4.15	0.006

NIHSS: National Institute of Health Stroke; IV tPA: intravenous recombinant tissue-type plasminogen activator; NLR: neutrophil-to-lymphocyte ratio; TICI: thrombolysis in cerebral infarction; ICH: intracerebral hemorrhage; OR: odds ratio; CI: confidence interval.

## Data Availability

The data used to support the findings of this study are available from the corresponding author.
